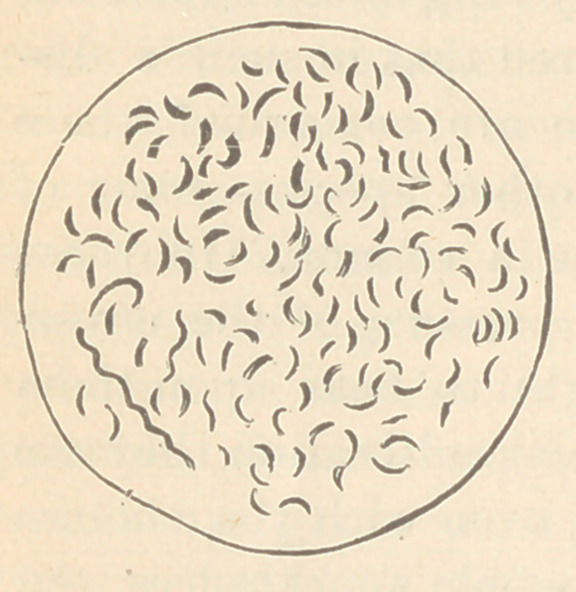# The Comma Bacillus of Asiatic Cholera

**Published:** 1885-04

**Authors:** Geo. W. Lewis

**Affiliations:** Berlin, Germany


### THE COMMA BACILLUS OF ASIATIC CHOLERA.

BY GEO. W. LEWIS, JR.

Sixty years ago, when but a single disease was recognized as the result of a minute organism slowly working its way from the surface towards the more vital parts of the human system, scientists and physicians alike would have scouted the idea, had it been proposed, that sixty years hence no less than seventy of the most deadly affections which man is heir to could be directly traceable to the activity of a lower animal life. This, however, is the case, and thanks to the progress of natural science and biology, we are now able to understand more fully that the history of life is a contention amid its various forms, and that all life of whatsoever sort has associated relations. Nor are these relations of a trivial and unimportant character, for there is generally found to be a means by which the welfare of all true life is promoted. This may show itself in either of two ways ; the lower forms may be destroyed in order to preserve the higher, or the lower life may serve as a means in disposing of that which would otherwise be injurious to our existence.

When we consider how intimate the relation is between the animal and the vegetable world, the theory of disease with all its complications becomes more and more evident, and with this gradual insight comes also a better understanding of the exact functions which these lower forms of life possess. To say that the vegetable life promoted the animal, and that the animal in its decay promoted the vegetable, formed the starting point from which all our present knowledge has been obtained. Add to this the marvelous revelations of the microscope of high magnifying power, and we have thus far ascertained the causative agent in a vast number of contagious diseases. That these diseases, moreover, are at least influenced if not caused by the presence of the organisms, is proved by the fact that their destruction in the living body causes not only a marked change, but also a very evident decrease in the intensity of the affection. It is, however, in many cases extremely difficult to find a remedy which will kill the organism and at the same time not injure the health of the patient. The power to resist strong reagents is very marked in some classes of micro-organisms, while in others the application of a very weak solution is sufficient to destroy their fatal tendencies.

The rapid advance which is being made in this department of medicine, has so far out-distanced the progress of rational therapeutics that a complete revival of the latter is needed in order to make these discoveries seem of any avail. The question has often been asked, "What is the use of all these so-called causative agents in disease, if a radical cure cannot be effected?" The answer is simply this : experience has shown that the healthy body, when attacked by disease, passes through certain changes, and the object was primarily to ascertain, if possible, the microscopic appearances which attended these changes. In so doing, a strong hope was entertained that some revelations might be made which would enable rational therapeutics to act with greater certainty. In a large number of diseases a high magnifying power revealed the presence of minute masses of protoplasm, each moving so rapidly that their characteristic form and properties could not be distinguished. Moreover, these masses of protoplasm seemed to be divided off into colonies, each occupying an almost circular position in the affected region. In come cases colonies presenting a very different appearance were noticed, thus showing the invasion of more than one species. Wherever several distinct species were detected, however, there was always one which seemed to predominate. This fact, once established, was sufficient to encourage the most sanguine to adopt any method which would enable them to study the form and function of the individual organism. The next step, therefore, was to apply some artificial coloring matter which would give a clear outline of the form, and at the same time place it in a stationary position. As a result of all these investigations, several classes of organisms have come to be recognized under the names of Bacilli, Micrococci, Spirilla, etc. Although a means of exterminating these foreign invaders into the human system has not in all cases been found as yet, it is to be hoped that active workers in the field of rational therapeutics will soon be able to devise some plan by which their satisfactory removal can be effected. Until that time is reached, however, the theory of their disease-causing property must not be despaired of, for time and experience have both shown that this is the only true method of becoming acquainted with the inner workings of the pathological condition; and the wonderful results which have already been achieved will undoubtedly spur on others to a greater activity.

Inasmuch as the object of this paper is to describe as lucidly as possible the comma bacillus of Asiatic cholera, to which the writer has given particular attention, reference to others of its class will only be made in order to render stronger the contrast between them. With regard to Micrococci and Spirilla, they are organisms of an entirely different nature, and require a separate treatment. It may be well to state for the benefit of those unacquainted with this subject, that the term Bacillus is applied to all forms of miscroscopic life which are characterized by a length two or three times as great as their width, or in other words an organism presenting, under a magnifying power of 1000 diameters, very much the appearance of a piece of thread one-sixteenth of an inch in length, and one forty-eighth of an inch in width. With the understanding, therefore, that the perfectly healthy body is possessed of an almost infinite variety of micro-organisms, and that it is only the invasion of a foreign organism which causes the disease, I wish to say a few words about the difficulty in distinguishing between the different kinds of bacilli.

With the exception of Dr. Koch's cholera bacillus, and the bacillus recently demonstrated by Dr. W. D. Miller, of Berlin, to be the cause of inflammation of the gums, it is almost impossible to distinguish between the various species of organisms known to us by this name as they appear under the microscope. This difficulty, however, is obviated by carrying out an artificial cultivation in some nourishing medium, such as food-gelatine, or bouillon, wherein the different varieties are easily distinguishable by their peculiar mode of growth. The cholera bacillus and the bacillus of inflamed gums both possess a marked curve resembling that of a comma, and for this reason can be readily recognized; but the bacilli of typhus fever, tuberculosis, gangrene and many other diseases, are so much alike that a safe diagnosis can only be made by artificially cultivating them. It may seem to many that this idea of reproducing an organism outside of the body for the sake of proving its identity, involves an exceedingly long and, in fact, unnecessary process ; but it must be remembered that in such a disease as cholera, where the intestines alone are concerned, there are, besides the comma bacillus, several other species, some of which appear to possess curves not unlike a comma. In these cases, however, the form is probably due to pressure of the cover-glass, or to some other force which is brought to bear upon them during the coloring process, for in an artificial cultivation they are invariably straight. It is better, therefore, even with the cholera bacillus, not to trust too much to the microscopic appearances. An artificial growth can easily be obtained in twenty-four hours, and will then put an end to all speculation and guess-work.

In saying that cholera is an affection exclusively of the intestines, I base the statement upon one hundred examinations made by Dr. Koch with a view of establishing this fact. He claims that in spite of the most careful investigation of all the other organs and of the blood, nothing was found which would lead one to suppose that the infectious material was to be found there. Moreover, only a comparatively small part of the intestines seemed to be the real seat of the disease, the ileum, including Peyers glands, being the part most affected. Upon examination of pieces of the intestine, and of its contents, there were found to be immense numbers of comma bacilli in the vicinity of the glands, thus causing the red and inflamed appearance around the edges; they were also found to have penetrated deeper into the tissues, forcing their way between the epithelium and the basement membrane. Behind these bacteria various other bacilli were noticed, some longer and thicker, others very short and thin. The characteristic curve, however, was wanting except where the comma bacilli were present, and no pathological change was detected except in their tracks. The comma bacilli are described as always being in advance of the non-pathogenic bacteria, the former forcing their way further in to prepare the path for the latter. This would seem to indicate beyond a doubt that they play an important part in the malady, and that without them the disease would in all probability be unknown.

It is difficult, in such a paper as this, to give a clear idea of how the comma bacillus looks under the microscope; indeed, to describe it in words would be almost impossible. Perhaps, however, the following cut may serve to illustrate its main characteristics. Occasionally two of the organisms are attached together, giving the appearance of the letter S; then again several, by means of the same process, form a wavy thread. This appearance has given rise to the theory that the comma bacillus is not a genuine bacillus, but only a detached form of a spirillum. It is not necessary, however, to discuss this question here; suffice it to say that Dr. Koch rather adheres to this belief.

Experiments have shown that the temperature most favorable to the growth and development of this organism range between 86° and 104° Fahrenheit. The growth is rapid, and a maximum is soon reached, after which a speedy decline sets in. This stage is characterized by a gradual wasting away, and a consequent loss of form. During their activity the proportion of comma bacilli to other intestinal bacteria has in some cases been estimated as high as ten to one. This, although not a trustworthy guide, indicates the tendency of all rival life ; that which is higher in quality must succumb to that which is greater in quantity. Another peculiarity of this microbe is that it dies very quickly after being dried. In this respect it closely resembles the spirillum, for as far as is known no other micro-organism is effectually exterminated from want of moisture.

Although comparatively little is known of the life history of the comma bacillus, we are nevertheless able to detect in it a striking relation to the cholera process. The fact that the organism reaches its fullest development in a single day, coincides with the almost phenomenal progress which is made in the disease in a similar time; then, too, the loss of form which quickly follows after it has reached the maximum growth, would seem to imply a corresponding loss of power. The excreta of persons who had recovered from a very evident attack of cholera were examined both before and after the disease had subsided, with a view of establishing the relationship which existed between the comma bacilli and the other intestinal bacteria. While the disease was at its height, the excreta contained enormous quantities of comma bacilli, with very few other bacteria. Later on, when a favorable change in the condition of the patients was noticed, examinations revealed a marked decrease in the former, and a corresponding increase of the latter. After complete recovery no comma bacilli were to be found.

Before closing this paper I desire to say that everything is in favor of regarding cholera as a germ disease. The fact that all attempts have failed to produce the cholera symptoms in animals, by inoculating comma bacilli into their systems, does not necessarily disprove the theory that the comma bacillus is the cause of cholera. Few people at the present time doubt that leprosy is caused by a specific organism in the shape of a bacillus, and yet it is impossible to produce that disease in animals by inserting the infectious material into their bodies. It simply shows that cholera, like leprosy, is an affection peculiar to the human race. On the other hand, we know of several diseases of animals which cannot be transferred to man; for example, rinderpest, and pneumonia of cattle. We meet here with a phenomenon widely spread in nature. Almost all parasites are restricted to one, or, at most, only a few classes of animals, which seem to act as their host. A striking illustration of this is to be found among tape-worms. Nearly every species of animal has its own special tape-worm, which can only be developed in that species.

It is now far from satisfactory to pronounce as to many diseases until their etiology has been thoroughly explored, and until, by the method of exclusion, we are able to say of this or that symptom, or of the aggregate of symptoms, that they do not result from the activity of a lower animal life. The same is true as we pass into the still broader field of plant life. When reference is made to the germ theory of disease, many at once take it for granted that the allusion is to some form of animal vitality. The fact is, however, that the great contention between life, disease and death, has to do with plants. Whether we speak of spore, of fungus, of bacteria, or of other low forms of existence, it is generally with botanical principles that we have to deal. Ardent workers in this department of science have demonstrated beyond a doubt, that there is a plant-world too small for the human eye, and that it possesses thousands of forms, each knowing its own soil. Some departure from the normal condition constitutes the chosen nourishment for each of these forms, and in their sudden and rapid growth we have the actual manifestation of various diseases. We have already singled out and classified this lower life enough to show that there is a disease botany of the future, as intricate as was that of the life botany of the vegetable world a hundred years ago. It demands a more minute study and intricate classification, and, because so imperceptible, will require a longer time for its development into a complete system.

BERLIN, GERMANY, *February* 8, 1885.

## Figures and Tables

**Figure f1:**